# Supplementary material for: In-depth single-cell analysis of translation-competent HIV-1 reservoirs identifies cellular sources of plasma viremia
Source: Nat Commun. 2021 Jun 17;12:3727. doi: 10.1038/s41467-021-24080-1 (PMC8211655; doi:10.1038/s41467-021-24080-1)
Supplement: Supplementary file 1 — Supplementary Information [file 41467_2021_24080_MOESM1_ESM.pdf]

Supplementary Information

In-depth single-cell analysis of translation-competent HIV-1 reservoirs identifies cellular sources of plasma viremia

Cole *et al.*

Supplementary Fig. 1: Frequency of p24+ cells after methanol fixation: linearity and comparison with PFA. a, b Linearity of the p24+ frequency with STIP-seq. J1.1 cells were serially diluted in Jurkat cells, fixed/permeabilized with methanol and stained with HIV p24-specific KC57 and 28B7 antibodies. **a** FACS dots plots of co-staining KC57/28B7 cells. **b** Linear regression of p24+ frequency from serially diluted J1.1 cells in Jurkat cells. Predicted curve is represented by the dashed line. **c** Comparison of p24+ frequency using PFA-based fixation vs. methanol-based fixation on CD4 T cells from 1 viremic and 4 ART-treated individuals (P5, P6 T1, P7 T1, P8 T1). Each participant is represented by a color-coded symbol. For statistical analysis, a two-sided matched-pairs Wilcoxon signed-rank test was used to compare frequencies. PFA = paraformaldehyde.

Supplementary Fig. 2: STIP-Seq decision tree. Step-by-step overview of the methodologies applied during STIP-Seq. Single sorted p24+ cells are subjected to whole genome amplification by multiple displacement amplification. Reactions are screened for successful amplification with a duplex qPCR (RPP30 reference gene, HIV LTR). Wells containing a cell with successfully amplified DNA are subjected to integration site analysis by integration site loop amplification, near full-length proviral sequencing with a 5- or 2-amplicon approach and TCR sequencing. WGA = whole genome amplification, LTR = long terminal repeat, ISLA = Integration Site Loop Amplification, NFL = Near full-length sequencing, TCR = T cell receptor.

Supplementary Fig. 3: Deletions in the 5'UTR, p17 gene and p24 gene of translation-competent proviruses. Graphical representation of the 5'UTR-p17-p24 region of proviruses recovered with STIP-Seq that had deletions spanning into p17/p24. Participant IDs are indicated on the left and the corresponding integration sites on the right side of each genome depiction. UTR = untranslated region.

Supplementary Fig. 4: p24-antibody fluorescence intensity comparison. Flow-cytometry fluorescence intensities (FI) for the KC57-FITC antibody and the 28B7-APC antibody were normalized by z-score (standard score) normalization (normalized FI = [FI - mean(FI all p24+ cells)] / SD(FI all p24+ cells)). **a** Correlation dot-plot of normalized KC57-FITC and 28B7-APC FI, colored by participant. A non-parametric Spearman rank correlation test was performed (rho=0.93, p = 0.000078). **b, c** Violin plots of normalized 28B7-APC and KC57-FITC FI of cells harboring a provirus with *gag* AUG deletion vs. a provirus with intact *gag* AUG. The mean FI is indicated by a horizontal line. A two-sided non-paired Wilcoxon signed-rank test was used to compare both populations (p=0.71 for 28B7-APC and p=0.70 for KC57-FITC). **d, e** Violin plots of normalized 28B7-APC and KC57-FITC FI of cells harboring a defective vs. intact provirus. The mean FI is indicated by a horizontal line. A two-sided non-paired Wilcoxon signed-rank test was used to compare both populations (p=0.70 for 28B7-APC and p=0.49 for KC57-FITC).

Supplementary Fig. 5: Comparison between FLIPS and STIP-Seq on 2 longitudinal samples from an ART-suppressed individual. Maximum-likelihood phylogenetic trees from near full-length proviral genomes generated with FLIPS and STIP-Seq. STIP-Seq and FLIPS were performed on CD4 T cells from an ART-suppressed individual (P5), at

two time points, 3 years apart. Bars depict the length of each proviral sequence, colored according to the NFL class. The blue arc depicts an identical proviral genome sequence that was found with both FLIPS and STIP-Seq.

Supplementary Fig. 6: Near-full length proviral genome class of unique versus clonal cells. Bar plots showing the proportions of proviruses with an internal deletion, PSI/MSD defect or intact among clonal infected cells vs. infected cells with a unique provirus. Cells from clonal populations were only counted once. A two-sided Fisher exact test was performed to compare both populations (p=0.99). NFL = near full-length, PSI = packaging signal, MSD = major splice donor.

Supplementary Fig. 7: Influence of methanol-fixation and PMA/ionomycin stimulation on memory subsets. Representative FACS dot plots showing the co-staining of cells with CD45RO and CD27 (gated on live CD4 T cells). Purified CD4 T cells were stimulated or not with PMA/ionomycin and were subjected to either paraformaldehyde or methanol fixation after 24h. The central/transitional memory T cell quadrant (CD45RO+ CD27+; TCM/TTM) is colored in blue, the effector memory T cell quadrant (CD45RO+ CD27-; TEM) in red, the naïve T cell quadrant (CD45RO- CD27+; TN) in green and the terminally differentiated memory T cell quadrant (CD45RO- CD27-; TTD) in purple. PMA = phorbol myristate acetate.

Supplementary Fig. 8: Longitudinal STIP-Seq analysis on ART-suppressed individual. Alluvial plots showing the memory phenotype of the host cell, the IS and the NFL class for each p24-producing cell from two longitudinal samples of participant P5 (3 years apart). Single p24+ sorted cells are represented on the y-axis of each plot. IS = integration site, NFL = near full-length class, TN = naïve T cell, TCM = central memory T cell, TTM = transitional memory T cell, TEM = effector memory T cell.

Supplementary Fig. 9: Gating strategy used in STIP-seq. Representative example of the gating strategy used in STIP-Seq following PMA/ionomycin stimulation of CD4 T cells obtained from an ART-suppressed individual. p24+ (KC57+/28B7+) were single cell sorted. Index cell sorting allowed for *post hoc* analysis of the memory phenotype of p24+ cells, by using the CD27 and CD45RO markers. Arrows indicate the sequential gating order. FSC = forward scatter, SSC = side scatter, TN = naïve CD4 T cell, TCM/TTM = central/transitional memory CD4 T cell, TEM = effector memory CD4 T cell, TTD = terminally differentiated CD4 T cell.

Supplementary Table 1: Clinical and virological characteristics of participants. Participant P5 was sampled at two timepoints during ART-suppression (3 years apart), indicated in the Timepoint column as 'First' and '+3 years' respectively. Participants P6 and P7 were sampled during ART-suppression and during analytical treatment interruption, indicated in the Timepoint column as 'T1' and 'T2' respectively. ART = antiretroviral therapy; VL = viral load.

Supplementary Table 2: Near-full length proviral PCRs. Summary of PCR success using the 5-amplicon, 2-amplicon and 4-amplicon PCR approaches for near full-length proviral sequencing. Green color = positive PCR result, Red color = negative PCR result, Grey color = not attempted. NFL = near full-length sequencing, LH = left half, RH = right half, Frag = fragment, NA = not available, HXB2 = subtype B HIV-1 reference genome.

Supplementary Table 3: Integration sites of p24+ cells. Integration sites were mapped to the GRCh38.p2 human genome reference assembly. Cancer-related genes are indicated with an asterisk. Chrom. = chromosome, NA = not applicable.

Supplementary Table 4: TCR sequences and predicted specificities of p24+ cells. TRBV/TRBJ usage and CDR3 amino acid sequence for each sorted p24+ cell. NA = not available.

Supplementary Table 5: Primers used for near full-length proviral sequencing. NFL = near full-length proviral sequencing, HXB2 = subtype B HIV-1 reference genome.

Supplementary Fig. 1

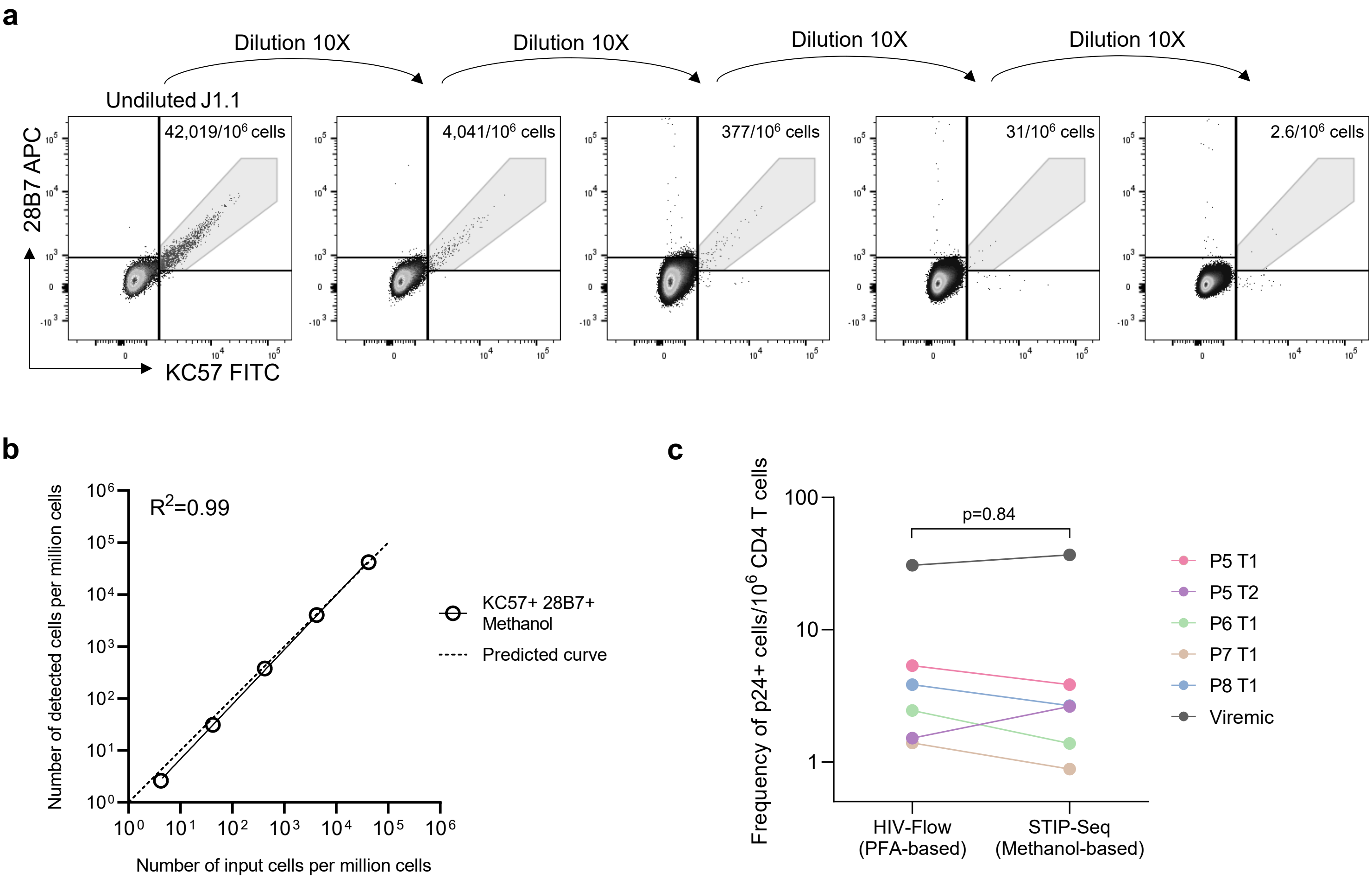

Supplementary Fig. 2

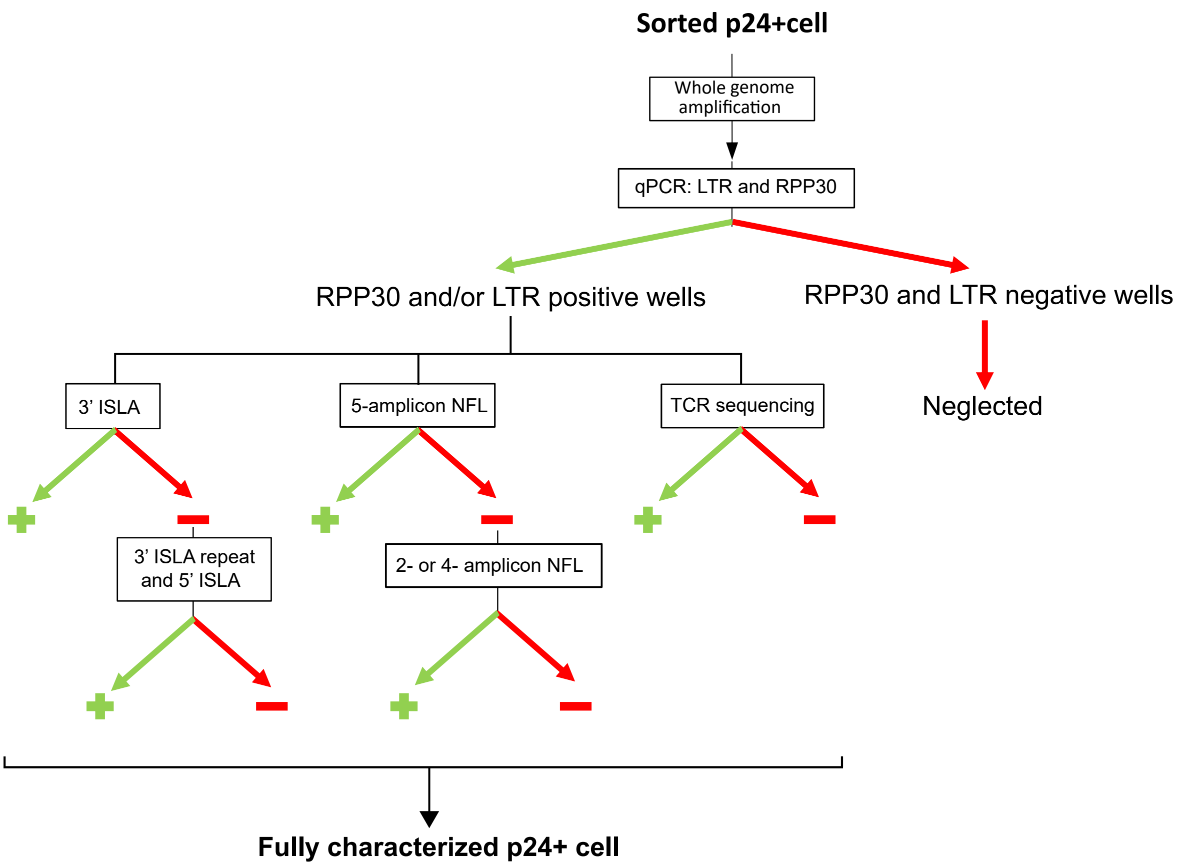

Supplementary Fig. 3

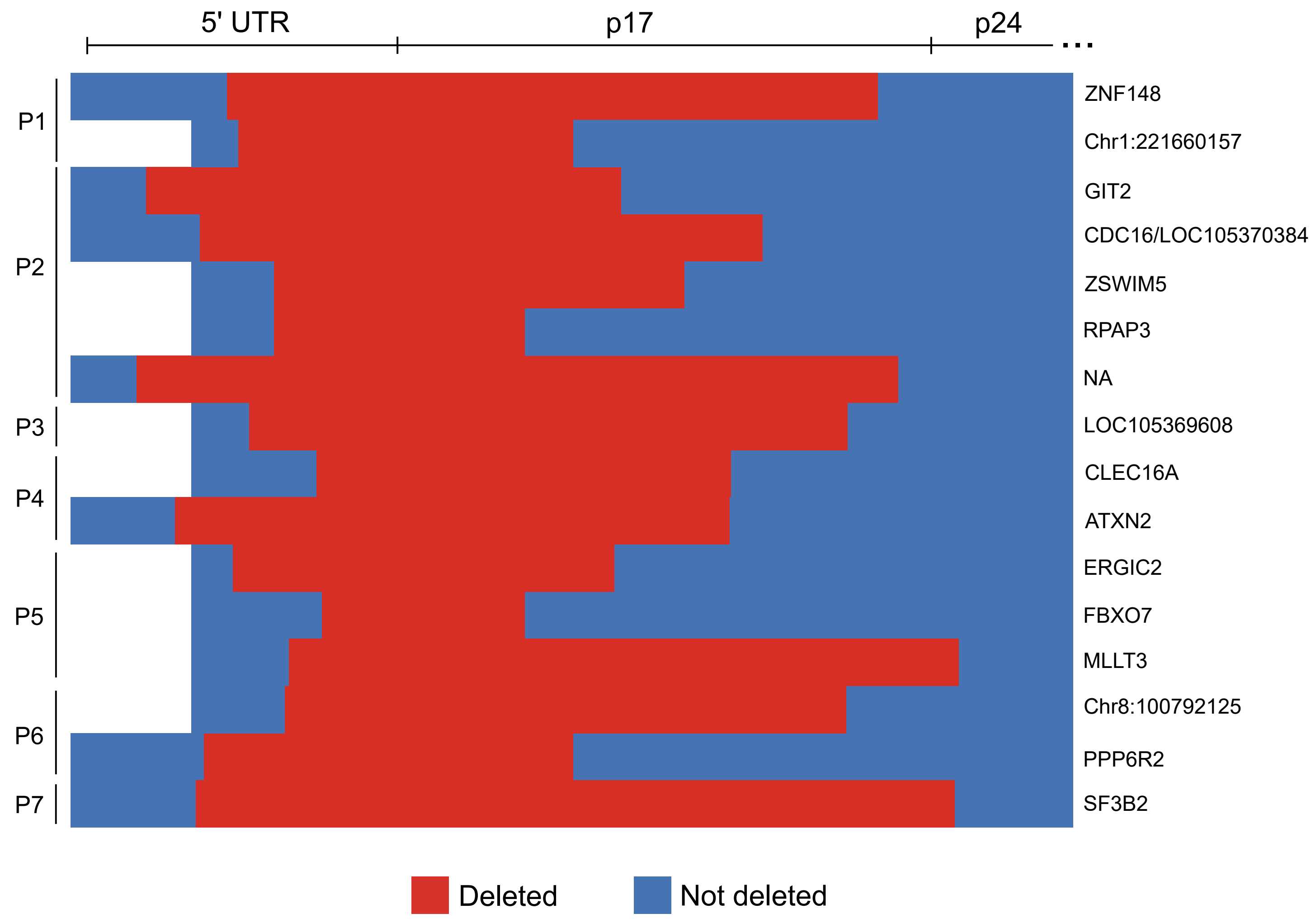

Supplementary Fig. 4

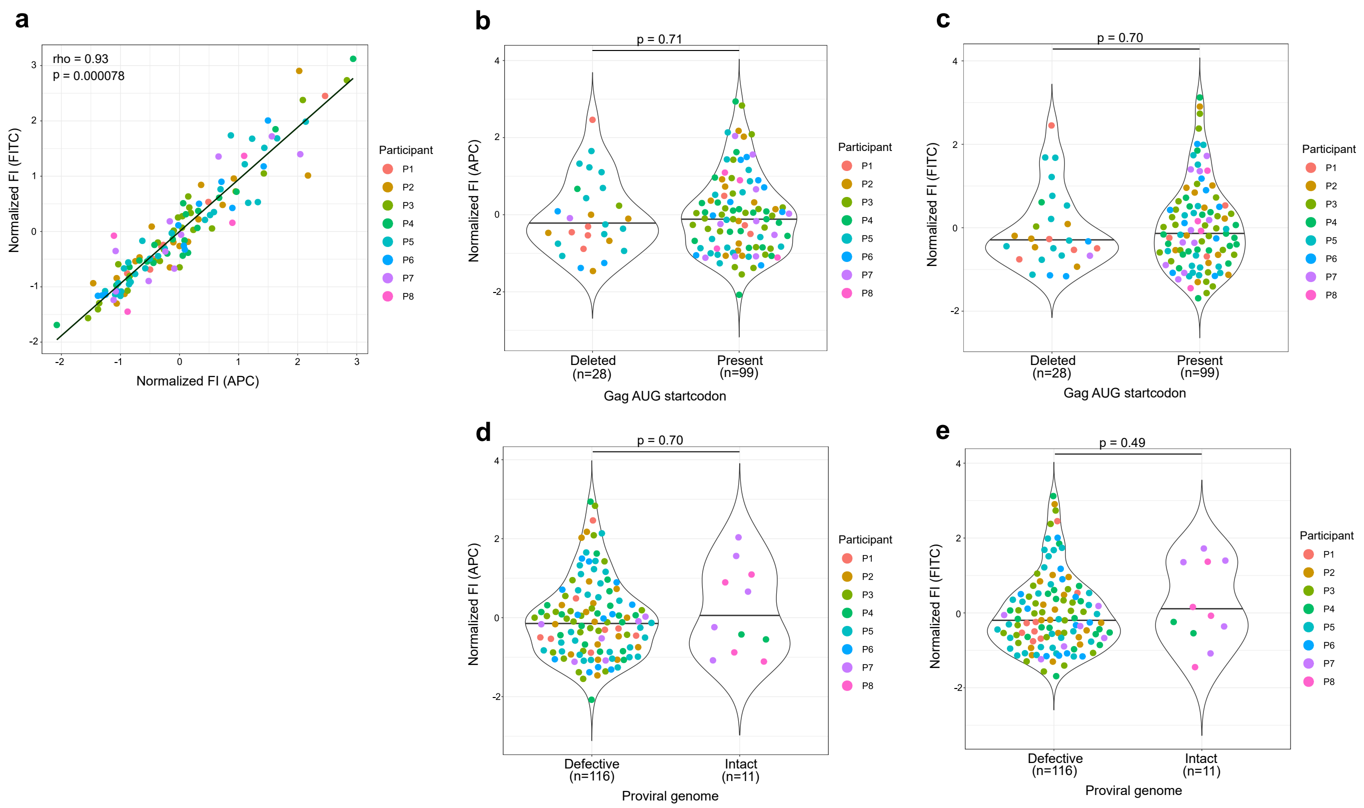

Supplementary Fig. 5

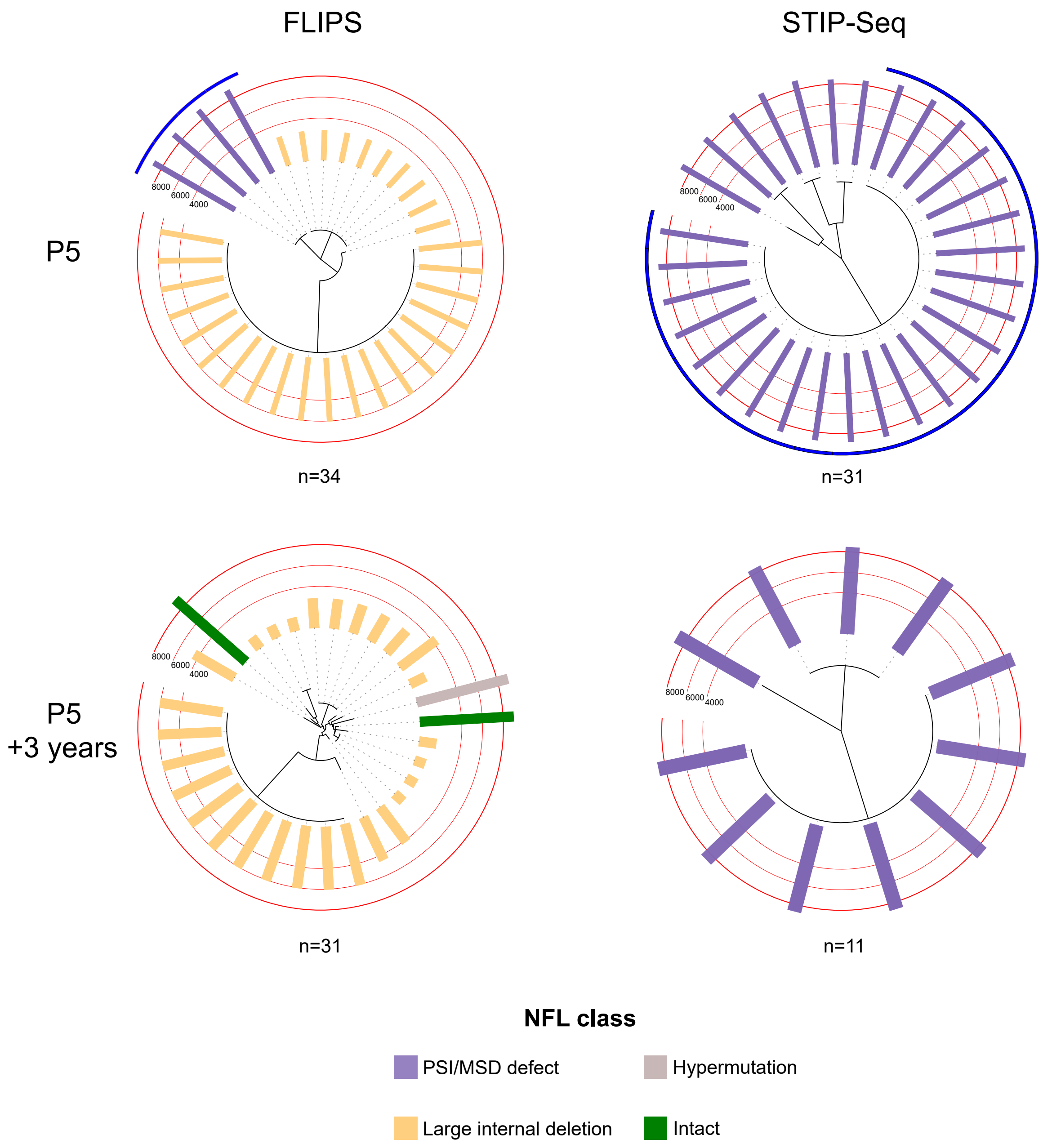

Supplementary Fig. 6

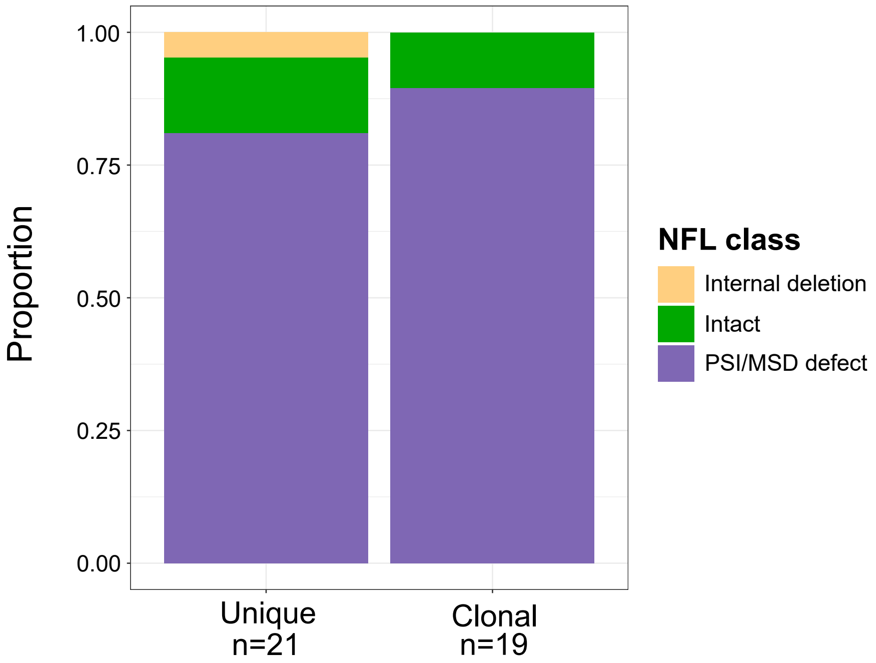

Supplementary Fig. 7

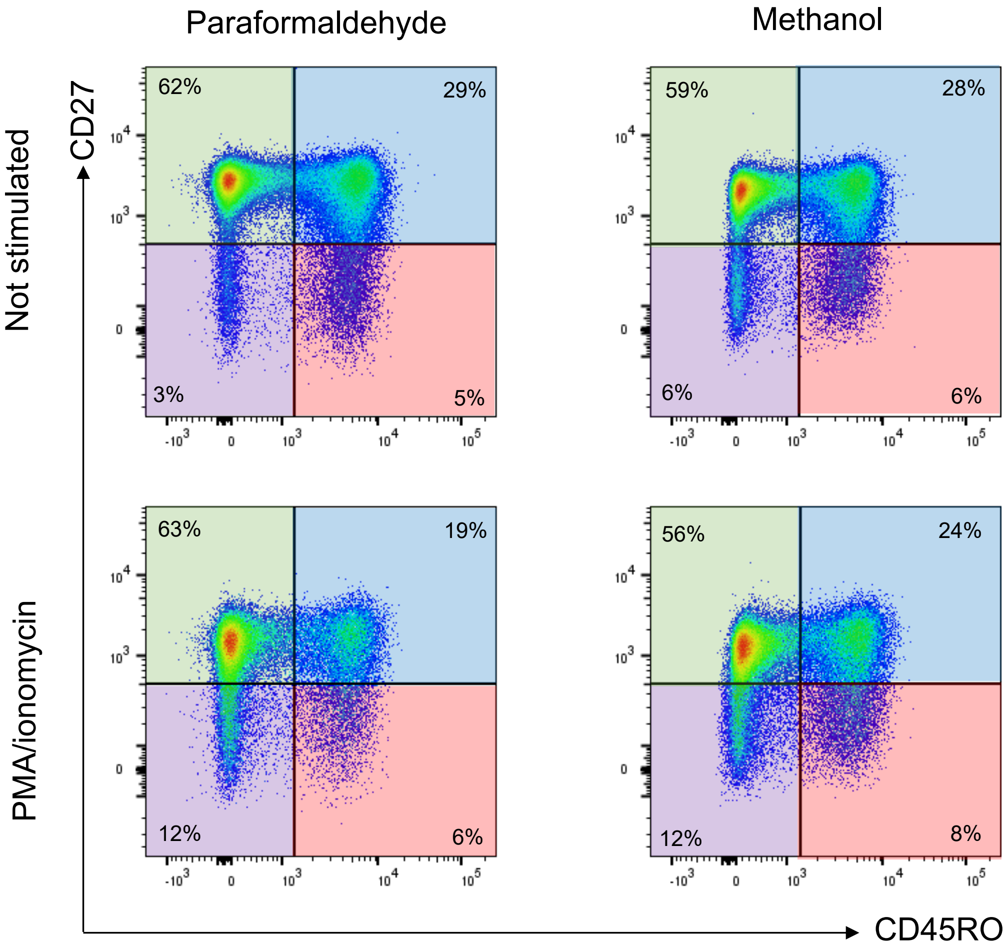

Supplementary Fig. 8

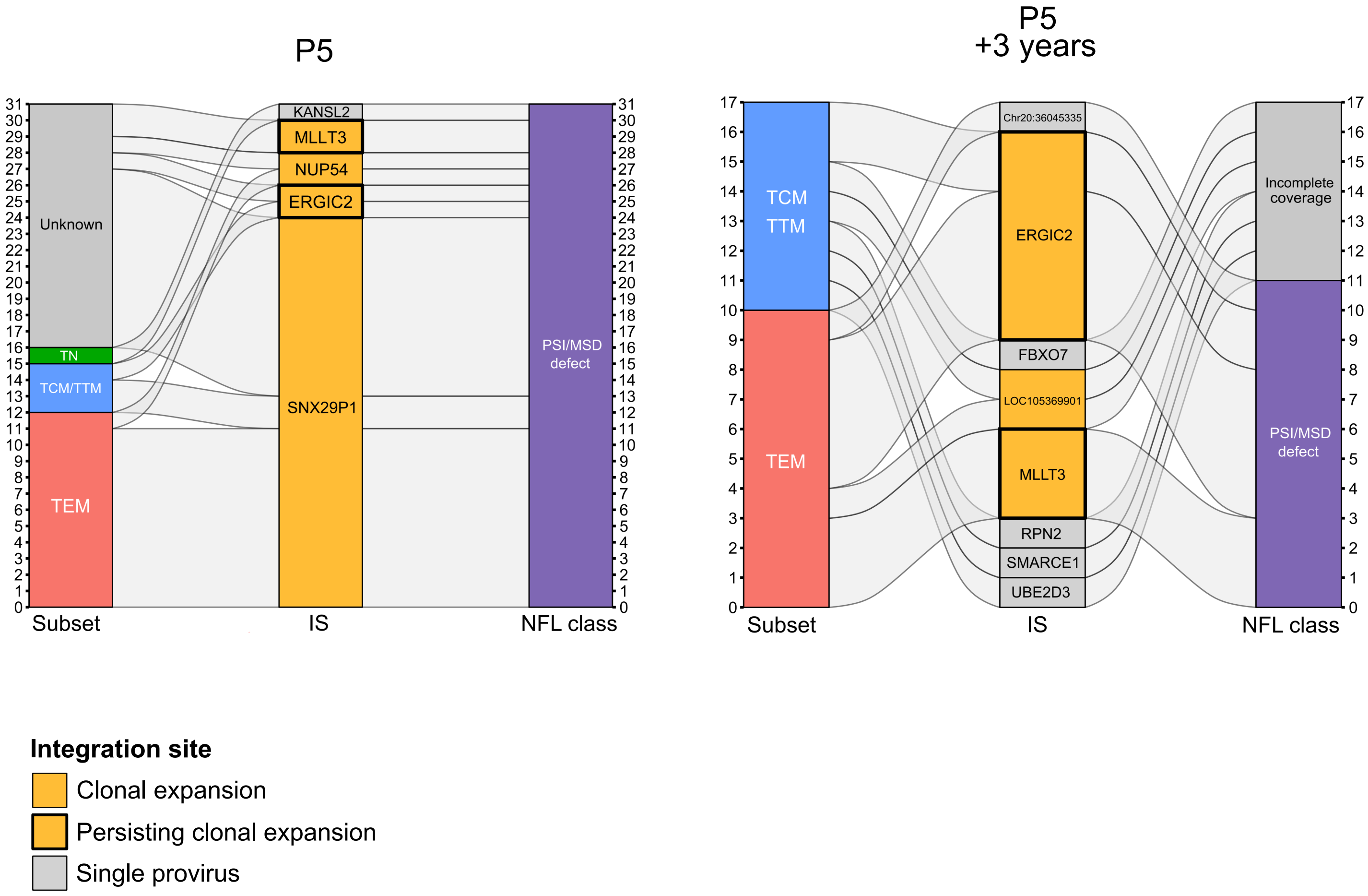

Supplementary Fig. 9

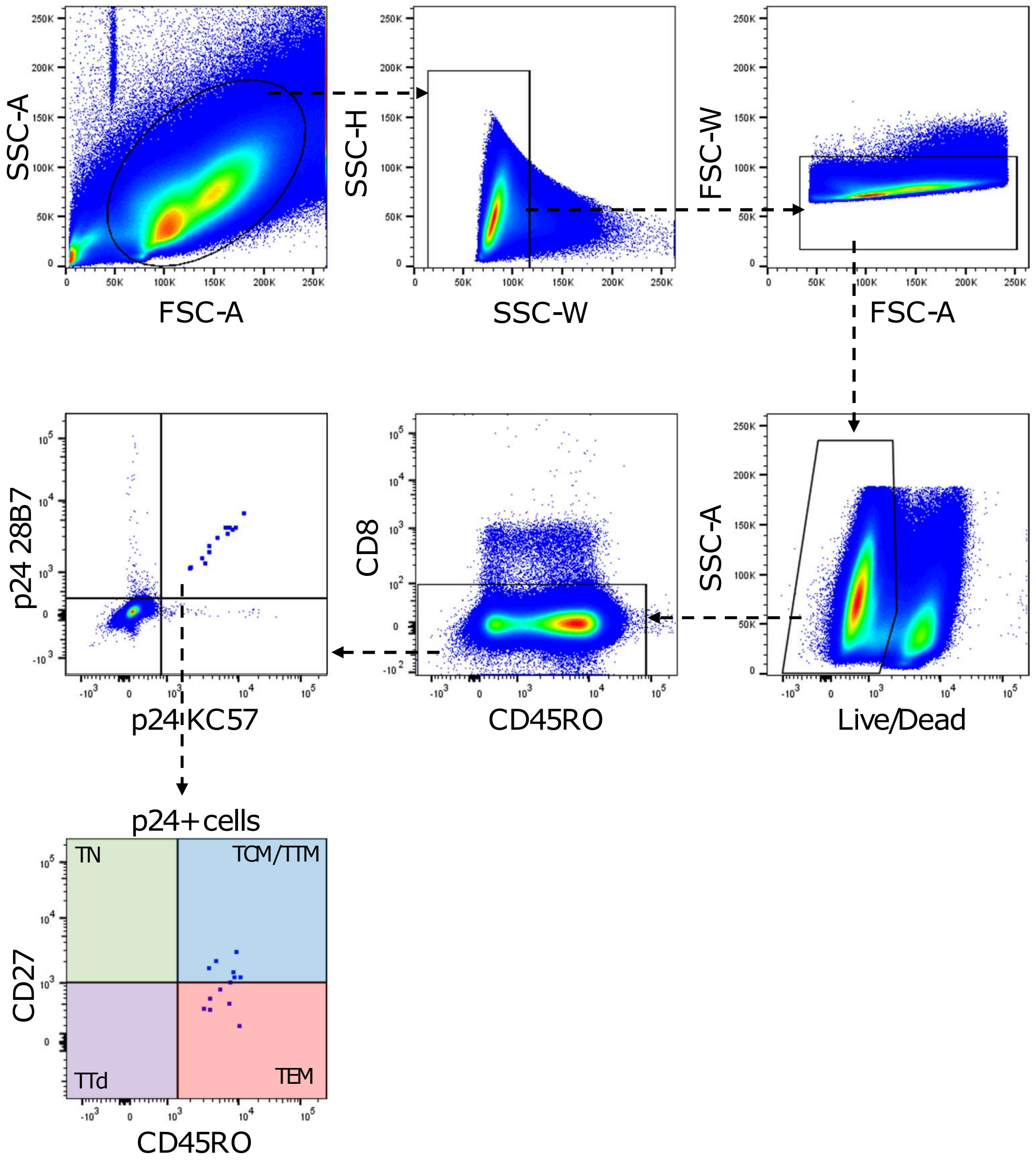

Supplementary Table 1

Participant ID	Timepoint	On/off ART	Time to ART initiation (years)	ART duration (years)	CD4 count (cells/µL)	CD4/CD8 ratio	VL at sampling (copies/ml plasma)	Median p24+ frequency (p24+ cells/million CD4 T cells)
P1	x	ON	6.5	16	601	0.7	<40	3.5
P2	x	ON	4.8	22	1076	1.9	<40	9.5
P3	x	ON	7.8	7	445	2.0	<40	4.2
P4	x	ON	0.6	20	911	1.0	< 20	2.9
P5	First	ON	0.4	14	293	0.3	< 20	3.8
P5	+ 3 years	ON	0.4	17	343	0.3	< 20	2.6
P6	T1	ON	3.0	11	736	0.6	< 20	1.4
P6	T2	OFF	3.0	11	701	0.7	< 20	1.8
P7	T1	ON	6.9	2	911	0.9	< 20	0.9
P7	T2	OFF	6.9	2	985	1.6	< 20	0.8
P8	T1	ON	0.4	3	714	1.3	< 20	2.7

Supplementary Table 2

Participant	Integration site	Number detected	5-amplicon NFL A1mod2 (638-2724)	5-amplicon NFL pol (2011-3798)	5-amplicon NFL C (3626-5980)	5-amplicon NFL A2 (5549-7760)	5-amplicon NFL B2 (7652-9610)	2-amplicon NFL Left half (581-5783)	2-amplicon NFL Right half (5088-9602)	4-amplicon NFL Frag 1 (634-3500)	4-amplicon NFL Frag 2 (1870-5248)	4-amplicon NFL Frag 3 (4133-7338)	4-amplicon NFL Frag 4 (6445-9632)
			Amplicon name (HXB2 coordinates)										
P1	ZNF148	4											
P1	Chr1:221660157	1											
P1	ADARB1	3											
P1	Chr9:61817567	1											
P1	STAT5B	1											
P2	ITGB1	10											
P2	NA	1											
P2	GALM*	1											
P2	KHDRBS1	1											
P2	CDC16/LOC105370384*	1											
P2	GIT2	1											
P2	MGAT4A	1											
P2	RSPRY1	1											
P2	ZSWIM5	1											
P2	AP2A2	1											
P2	NSD1	1											
P2	RASA3	1											
P2	CPEB4	1											
P2	MAN2A1	1											
P2	RPAP3	1											
P3	TNRC6B	16											
P3	LOC105369608	1											
P3	STAT5B	4											
P3	CD200R1	3											
P3	Chr2:54321973*	1											
P3	THOC5*	1											
P4	CLEC16A	1											
P4	CMAHP	1											
P4	FCGRT	1											
P4	Chr17:8974901	8											
P4	ZNF274	1											
P4	DNAH6	1											
P4	CD27-AS1	1											
P4	PLCG1	1											
P4	CPNE1	1											
P4	USP12/RBBP8P2	2											
P4	NUTM2F/HIATL1	2											
P4	Chr9:136929821	1											
P4	ATXN2	1											
P5	SNX29P1/P2	24											
P5	MLLT3	5											
P5	ERGIC2	8											
P5	KANSL2	1											
P5	NUP54	2											
P5	UBE2D3	1											
P5	FBXO7	1											
P5	SMARCE1	1											
P5	RPN2	1											
P5	LOC105369901	2											
P5	Chr20:36045335	1											
P6	PPP6R2	1											
P6	STAT5B	12											
P6	Chr8:100792125	4											
P6	VMP1	3											
P6	CIT	1											
P7	Chr17:77978920	3											
P7	Chr17:7545670	5											
P7	KCNA3	7											
P7	SF3B2	1											
P7	NA	1											
P7	VMP1	2											
P8	SMG1P2	6											

NA = not available
*Sequencing failed

Supplementary Table 3

Participant	Chrom.	Position	Strand	Gene	Orientation	Number detected
P1	3	125291341	-	ZNF148	Same	4
P1	1	221660157	-	NA	NA	1
P1	21	45087397	+	ADARB1	Opposite	3
P1	9	61817567	-	NA	NA	1
P1	17	42253537	+	STAT5B*	Opposite	1
P2	10	32919286	+	ITGB1	Opposite	10
P2	2	38669512	-	GALM	Opposite	1
P2	1	32030557	-	KHDRBS1	Opposite	1
P2	13	114258553	-	CDC16/LOC105370384	Opposite/Same	1
P2	12	109948596	+	GIT2	Opposite	1
P2	2	98720877	+	MGAT4A	Opposite	1
P2	16	57192144	+	RSPRY1	Same	1
P2	1	45187297	+	ZSWIM5	Opposite	1
P2	11	941911	-	AP2A2	Opposite	1
P2	5	177176435	+	NSD1*	Same	1
P2	13	114093356	+	RASA3*	Opposite	1
P2	5	173905709	-	CPEB4*	Opposite	1
P2	5	109851902	+	MAN2A1	Same	1
P2	12	47677422	+	RPAP3	Opposite	1
P3	22	40309709	+	TNRC6B	Same	16
P3	12	3952756	-	LOC105369608	Same	1
P3	17	42258445	+	STAT5B*	Opposite	4
P3	3	112951052	+	CD200R1	Opposite	3
P3	2	54321973	-	NA	NA	1
P3	22	29509957	+	THOC5*	Opposite	1
P4	16	11119179	-	CLEC16A	Opposite	1
P4	6	25130744	+	CMAHP	Opposite	1
P4	19	49517975	-	FCGRT	Opposite	1
P4	17	8974901	+	NA	NA	8
P4	19	58195268	-	ZNF274	Opposite	1
P4	2	84652442	-	DNAH6	Opposite	1
P4	12	6439170	+	CD27-AS1	Opposite	1
P4	20	41167601	-	PLCG1*	Opposite	1
P4	20	35637188	+	CPNE1	Opposite	1
P4	13	27077455	-	USP12/RBBP8P2	Same/Opposite	2
P4	9	94413057	+	NUTM2F/HIATL1	Same/Opposite	2
P4	9	136929821	-	NA	NA	1
P4	12	111484919	-	ATXN2	Same	1
P5	16	21383557	-	SNX29P1/P2	Opposite	24
P5	9	20608766	+	MLLT3*	Opposite	5
P5	12	29347700	+	ERGIC2	Opposite	8
P5	12	48680541	+	KANSL2	Opposite	1
P5	4	76124580	-	NUP54	Same	2
P5	4	102812614	+	UBE2D3	Opposite	1
P5	22	32494019	-	FBXO7	Opposite	1
P5	17	40641871	+	SMARCE1*	Opposite	1
P5	20	37227689	+	RPN2	Same	1
P5	12	91925519	+	LOC105369901	Opposite	2
P5	20	36045335	+	NA	NA	1
P6	22	50358570	-	PPP6R2	Opposite	1
P6	17	42264648	+	STAT5B*	Opposite	12
P6	8	100792125	+	NA	NA	4
P6	17	59803733	-	VMP1*†	Opposite	3
P6	12	119761801	-	CIT†	Same	1
P7	17	77978920	-	NA	NA	3
P7	17	7545670	-	NA	NA	5
P7	1	110655131	-	KCNA3	Same	7
P7	11	66064686	-	SF3B2	Opposite	1
P7	17	59803547	-	VMP1*	Opposite	2
P8	16	29558266	-	SMG1P2	Same	6

†Only retrieved at T2, during analytical treatment interruption

NA = not applicable

Supplementary Table 4

Participant	Integration site	Number detected	TRBV	CDR3 (AA)	TRBJ	Specificity
P1	ZNF148	4	TRBV7-3	GRNQPQHF	TRBJ1-5	NA
P1	Chr1:221660157	1	TRBV18	CASSPGGLAETQFF	TRBJ2-6	NA
P1	ADARB1	3	TRBV27	CASSFFLNNHPPHF	TRBJ1-5	NA
P1	Chr9:61817567	1	TRBV7-9	CASSQGDGYGYTF	TRBJ1-2	*M. tuberculosis*
P1	STAT5B	1	TRBV9	CASSVVNRYAETQFF	TRBJ2-5	NA
P2	ITGB1	9	TRBV20-1	CSAGPSNQPQHF	TRBJ1-5	NA
P2	NA	1	NA	NA	NA	NA
P2	GALM	1	TRBV3-1	CASSQVTSGGARETHFF	TRBJ2-5	NA
P2	KHDRBS1	1	TRBV5-5	CASSSRTGGNEQFF	TRBJ2-1	NA
P2	CDC16/LOC105370384	1	NA	NA	NA	NA
P2	GIT2	1	TRBV5-4	CASSLQGASYNEQFF	TRBJ2-1	NA
P2	MGAT4A	1	TRBV20-1	CSARVRTSGYEQYF	TRBJ2-7	NA
P2	RSPRY1	1	TRBV5-4	CASSLVGAPPLNTEAFF	TRBJ1-1	NA
P2	ZSWIM5	1	TRBV29-1	CSVLPGQTEAFF	TRBJ1-1	NA
P2	AP2A2	1	NA	NA	NA	NA
P2	NSD1	1	TRBV30	CAWSARTDTQYF	TRBJ2-3	NA
P2	RASA3	1	NA	NA	NA	NA
P2	CPEB4	1	NA	NA	NA	NA
P2	MAN2A1	1	NA	NA	NA	NA
P2	RPAP3	1	TRBV14	CASSKGLAGGVTTDTQYF	TRBJ2-3	NA
P3	TNRC6B	16	TRBV5-1	CASSLEAGGNHPLF	TRBJ1-3	NA
P3	LOC105369608	1	NA	NA	NA	NA
P3	STAT5B	4	TRBV4-2	CASSQDEGYGYTF	TRBJ1-2	Influenza
P3	CD200R1	3	TRBV9	CASSPQGLNTEAFF	TRBJ1-1	CMV, influenza, *M. tuberculosis*
P3	Chr2:54321973	1	TRBV15	CATSRAQSNEKLFF	TRBJ1-4	NA
P3	THOC5	1	NA	NA	NA	NA
P4	CLEC16A	1	NA	NA	NA	NA
P4	CMAHP	1	TRBV7-9	CASSPGTYNSPLHF	TRBJ1-6	*M. tuberculosis*
P4	FCGRT	1	TRBV20-1	CSAQGPQGPQHF	TRBJ1-5	NA
P4	Chr17:8974901	8	TRBV20-1	CSARVRDRPYEQYF	TRBJ2-7	*M. tuberculosis*
P4	ZNF274	1	TRBV15	CATGTLAGRTLNTEAFF	TRBJ1-1	NA
P4	DNAH6	1	NA	NA	NA	NA
P4	CD27-AS1	1	NA	NA	NA	NA
P4	PLCG1	1	TRBV7-9	CASSQGSGEGETQFF	TRBJ2-5	NA
P4	CPNE1	1	NA	NA	NA	NA
P4	USP12/RBBP8P2	2	TRBV11-2	CASRRNAGTSDEQYF	TRBJ2-7	NA
P4	NUTM2F/HIATL1	2	TRBV19	CASSIGQGSNEELFF	TRBJ1-4	NA
P4	Chr9:136929821	1	TRBV6-1	CASSLKPRGANYGYYF	TRBJ1-2	NA
P4	ATXN2	1	NA	NA	NA	NA
P5	SNX29P1/P2	24	TRBV7-9	CASSRYRGR#TEAFF	TRBJ1-1	NA
P5	MLLT3	5	NA	NA	NA	NA
P5	ERGIC2	8	TRBV11-2	CASSLDGDSPLPF	TRBJ1-6	NA
P5	KANSL2	1	TRBV18	CASSPEVGSIGEQYF	TRBJ2-7	NA
P5	NUP54	2	TRBV20-1	CSADLGHRYPLF	TRBJ2-3	NA
P5	UBE2D3	1	NA	NA	NA	NA
P5	FBXO7	1	NA	NA	NA	NA
P5	SMARCE1	1	TRBV5-1	CASSSPGQGYEQYF	TRBJ2-7	NA
P5	RPN2	1	TRBV6-5	CASRRKGGRWDTTAFF	TRBJ1-1	NA
P5	LOC105369901	2	NA	NA	NA	NA
P5	Chr20:36045335	1	TRBV6-6	CAASSGGVLPPHFF	TRBJ1-6	NA
P6	PPP6R2	1	TRBV6-1	CASSEGWDDQQPPHFF	TRBJ1-4	NA
P6	STAT5B	12	TRBV19	CASSIAGRAFNASTF	TRBJ1-2	NA
P6	Chr8:100792125	4	TRBV2	CASSPNRGRGYTF	TRBJ1-2	CMV
P6	VMP1	3	NA	NA	NA	NA
P6	CIT	1	NA	NA	NA	NA
P7	Chr17:77978920	3	TRBV28	CATSFCGGRAADVLYF	TRBJ2-4	NA
P7	Chr17:7545670	5	TRBV5-5	CASSLGAGTGGTYGYTF	TRBJ1-2	NA
P7	KCNA3	7	TRBV7-2	CASSLKTGGYEQYF	TRBJ2-7	*M. tuberculosis*
P7	SF3B2	1	TRBV12-3	CASSLRDAAVAFF	TRBJ1-1	NA
P7	NA	1	TRBV5-4	CASSSKRGSTDTQYF	TRBJ2-3	*M. tuberculosis*
P7	VMP1	2	TRBV28	CASRPRRTYCYYF	TRBJ2-7	NA
P8	SMG1P2	6	TRBV18	CASSPEVGSIGEQYF	TRBJ2-7	NA

NA = not available

Supplementary Table 5

Assay	Amplicon	HXB2 coordinates	Forward/reverse	Round	Primer	Sequence (5' to 3')
RPP30 qPCR	RPP30	NA	Forward	1	Forward	AGATTTGGACCTGCGAGCG
			Reverse	1	Reverse	GAGCGGCTGTCTCCACAAGT
			Probe	1	Probe	TTCTGACCTGAAGGCTCTGCGCG
ISLA 3'end		8948	Forward	1	up3.2	CCAATGCTGATTGTGCCTGGCTAGAAGCA
		NA	Forward	2	deca1.u5	TCAAGTAGTGTGTGCCCGTCTGTNNNNNNNNNN
		9553	Forward	3	RF2	AGACCAGATCTGAGCCTGGGAGCTCTCTG
		9595	Forward	4	RF1	CCCACTGCTTAAGCCTCAATAAAGCTTGCCTTG
		9626	Forward	5	1.U5	TGAGTGCTTCAAGTAGTGTGTGCCCGTCTGT
		9647	Forward	6	2.U5	GCCCGTCTGTTGTGTGACTCTGGTAACTAGAGAT
ISLA 5'end		651	Reverse	1	UTR.629.R	CCCTGTTCGGGCGCCACTGCTA
		NA	Reverse	2	decaU3R.3	GTTCTGCCAATCAGGGAAGTAGCCTTGTGTGTNNNNNNNNNN
		160	Reverse	3	U3R.1	GGCTCAACTGGTACTAGCTTGAAGCACCATCCAAAG
		118	Reverse	4	U3R.2	GGATATCTGATCCCTGGCCCTGGTGTGTAGTT
		89	Reverse	5	U3R.3	GTTCTGCCAATCAGGGAAGTAGCCTTGTGTGT
		51	Reverse	6	U3R.4	CCCACAGATCAAGGATATCTTGTCT
5-amplicon NFL	A1mod2	623-3333	Forward	1	U5-623F	AAATCTCTAGCAGTGGCGCCCGAACAG
			Reverse	1	NE1	CCACTAACTTCTGTATGTCATTGACAGTCCAGCT
		638-2724	Forward	2	U5-638F	GCGCCCGAACAGGGACYTGAAARCGAAAG
			Reverse	2	ProC-	GAGTATTGTATGGATTTTCAGGCCCAAT
	pol	1981-3859	Forward	1	5CP1	GAAGGGCACACAGCCAGAAATTGCAGGG
			Reverse	1	RT3.1	GCTCCTACTATGGGTTCTTTCTCTAACTGG
		2011-3798	Forward	2	2.5	CCTAGGAAAAAGGGCTGTTGGAAATGTGG
			Reverse	2	RT3798R	CAAACTCCCACTCAGGAATCCA
	C	3597-6004	Forward	1	RT3597mixF	AAAACAGGAAARTATGCAA
			Reverse	1	SC05R	AGCTCTTCGTCGCTGTCTCCGCTT
		3626-5980	Forward	2	RT3626F	TGCCCACACTAATGATGTAA
			Reverse	2	SC02R	CTTCCTGCCATAGGAGATGCCTA
	A2	5450-7817	Forward	1	VP5450F	CAGGACATAACAAGGTAGGATC
			Reverse	1	CO602	GCCCATAGTGCTTCCTGCTGCTCCCAAGAACC
		5549-7760	Forward	2	VP5549F	AGAGGATAGATGGAACAAGCCCCAG
			Reverse	2	V3CR	TGCTCTTTTTTCTCTCTSCACCACT
	B2	7626-9628	Forward	1	GP41Fo	TTCAGACCTGGAGGAGGAGATAT
			Reverse	1	3LTRi	TCAAGGCAAGCTTTATTGAGGCTTAA
		7652-9610	Forward	2	GP41Fi	GGACAATTGGAGAAGTGAATTAT
			Reverse	2	3UTRi	AGGCTTAAGCAGTGGGTTCCCTAG
2-amplicon NFL	Left half	544-5968	Forward	1	F544	TTAAGCCTCAATAAAGCTTGCCTTGAG
			Reverse	1	R5968	TGTCTYCKCTTCTTCCTGCCATAG
		581-5783	Forward	2	F581	GTGTGCCCGTCTGTTGTGTGACTC
			Reverse	2	R5783	AATGCCTATTCTGCTATGTYGACACC
	Right half	5066-9665	Forward	1	F5066alt1	TATGGAAAACAGATGGCAGGTGMTGRT
			Reverse	1	R9665	GTCTGAGGGATCTCTAGWTACCAGA
		5088-9602	Forward	2	F5088alt1	GATTGTGTGGCARGTAGACAGRATG
			Reverse	2	R9602	CAAGGCAAGCTTTATTGAGGCTTAAS
4-amplicon NFL	NFL	623-9662	Forward	1	BLouterF	AAATCTCTAGCAGTGGCGCCCGAACAG
			Reverse	1	BLouterR	TGAGGGATCTCTAGTTACCAGAGTC
	Frag 1	634-3500	Forward	2	634(+)	AGTGGCGCCCGAACAGGGAC
			Reverse	2	3500(-)	CTATTAAGTATTTTGATGGGTCATAA
	Frag 2	1870-5248	Forward	2	1870(+)	GAGTTTTGGCTGAGGCAATGAG
			Reverse	2	5248(-)	TCTCCTGTATGCAGACCCCA
	Frag 3	4133-7338	Forward	2	4133(+)	GGAAAAGGTCTACCTGGCATG
			Reverse	2	E125(-)	CAATTTCTGGGTCCCCTCCTGAGG
	Frag 4	6445-9632	Forward	2	E30(+)	GTGTACCCACAGACCCCAGCCCACAAG
			Reverse	2	R-519(-)	GCACTCAAGGCAAGCTTTATTGAGGCTTA
FLIPS	Round 1	623-9662	Forward	1	BLouterF	AAATCTCTAGCAGTGGCGCCCGAACAG
			Reverse	1	BLouterR	TGAGGGATCTCTAGTTACCAGAGTC
	Round 2	646-9650	Forward	2	275F	ACAGGGACCTGAAAGCGAAAG
			Reverse	2	280R	CTAGTTACCAGAGTCACACAACAGACG

NA = not applicable

Supplementary Table 5 (cont.)

Assay	Amplicon	HXB2 coordinates	Forward/reverse	Round	Primer	Sequence (5' to 3')
TCR		NA	Forward	1	VB2	GTAAAACGACGGCCAGTATACTTCTATTGGTACAGACAAATCTTGG
		NA	Forward	1	VB3	GTAAAACGACGGCCAGTCTATGTATTGGTATAAACAGGACTCTAAG
		NA	Forward	1	VB4	GTAAAACGACGGCCAGTCAYARSGCTATGTATTGGTACAAGC
		NA	Forward	1	VB5/9	GTAAAACGACGGCCAGTCACTGTGTCCTGGTACCAACAG
		NA	Forward	1	VB6	GTAAAACGACGGCCAGTTACATGTACTGGTATCGACAAGACC
		NA	Forward	1	VB7	GTAAAACGACGGCCAGTTACCCTTTATTGGTACCGACAGAGCCTGG
		NA	Forward	1	VB11	GTAAAACGACGGCCAGTCTTTACTGGTACCGGCAGAWCYTGG
		NA	Forward	1	VB12	GTAAAACGACGGCCAGTTTTTCTGGTACAGACAGACCATGATG
		NA	Forward	1	VB13	GTAAAACGACGGCCAGTCACTGTCTACTGGTACCAGCAGG
		NA	Forward	1	VB14	GTAAAACGACGGCCAGTTGGACATGATAATCTTTATTGGTATCGAC
		NA	Forward	1	VB15	GTAAAACGACGGCCAGTCATGTACTGGTACCAGCAGAAGTC
		NA	Forward	1	VB16	GTAAAACGACGGCCAGTGTTATGTTTTTTGGTACCAACAGGTCC
		NA	Forward	1	VB17	GTAAAACGACGGCCAGTCATGTTTGTTCACTGGTACCGACAGAATC
		NA	Forward	1	VB18	GTAAAACGACGGCCAGTAGTCATGTTTACTGGTATCGGCAGC
		NA	Forward	1	VB19	GTAAAACGACGGCCAGTTGCCATGTACTGGTACCGACAG
		NA	Forward	1	VB20	GTAAAACGACGGCCAGTCACAACTATGTTTTGGTATCGTCAG
		NA	Forward	1	VB21	GTAAAACGACGGCCAGTTAGTTATGTTTACTGGTATCATAAGACGC
		NA	Forward	1	VB23	GTAAAACGACGGCCAGTATACTTTTTGTTTATTGGTATCAACAGAATCAG
		NA	Forward	1	VB24	GTAAAACGACGGCCAGTATGTACTGGTATCGACAAGACCC
		NA	Forward	1	VB25	GTAAAACGACGGCCAGTTGACAAAATGTACTGGTATCAACAAGATC
		NA	Forward	1	VB29	GTAAAACGACGGCCAGTTGATGTTCTGGTACCGTCAGCAAC
		NA	Forward	1	VB30	GTAAAACGACGGCCAGTCAACCTATACTGGTACCGACAGG
		NA	Reverse	1	JB1-1	CAGGAAACAGCTATGACCAACTGTGAGTCTGGTGCCTTGTCCAAAG
		NA	Reverse	1	JB1-2	CAGGAAACAGCTATGACAACCTGGTCCCCGAACCGAAGG
		NA	Reverse	1	JB1-3	CAGGAAACAGCTATGACAACAGTGAGCCAACTTCCCTCTCCAAAATA
		NA	Reverse	1	JB1-4	CAGGAAACAGCTATGACCAGAGAGCTGGGTTCCACTGCCAAAAAACA
		NA	Reverse	1	JB1-5	CAGGAAACAGCTATGACAGAGTCGAGTCCCATCACCAAAATGC
		NA	Reverse	1	JB1-6	CAGGAAACAGCTATGACCTGGTCCCATTCCCAAAGTGGAGG
		NA	Reverse	1	JB2-1	CAGGAAACAGCTATGACAGCCGTGTCCCTGGCCCGAAGAAC
		NA	Reverse	1	JB2-2	CAGGAAACAGCTATGACCGTTTTTTGGAGAAGGCTCTAGGCTGACC
		NA	Reverse	1	JB2-3	CAGGAAACAGCTATGACCAGCCGGGTGCCTGAGCCAAAATAC
		NA	Reverse	1	JB2-4	CAGGAAACAGCTATGACCGGGTCACGGCGCCGAAGTAC
		NA	Reverse	1	JB2-5	CAGGAAACAGCTATGACAGCCGCGTCCTGGCCCGAAG
		NA	Reverse	1	JB2-6	CAGGAAACAGCTATGACCTGCCGGCCCCGAAAGTCAGG
		NA	Reverse	1	JB2-7	CAGGAAACAGCTATGACCCTGGTGCCCGACCCGAAG
		NA	Forward	2	M13F	GTAAAACGACGGCCAGT
		NA	Reverse	2	M13R	CAGGAAACAGCTATGAC

NA = not applicable
