# Supplementary material for: In-depth single-cell analysis of translation-competent HIV-1 reservoirs identifies cellular sources of plasma viremia
Source: Nat Commun. 2021 Jun 17;12:3727. doi: 10.1038/s41467-021-24080-1 (PMC8211655; doi:10.1038/s41467-021-24080-1)
Supplement: Supplementary file 3 — Reporting Summary [file 41467_2021_24080_MOESM3_ESM.pdf]

nature research

Corresponding author(s): Linos Vandekerckhove

Last updated by author(s): May 17, 2021

Reporting Summary

Nature Research wishes to improve the reproducibility of the work that we publish. This form provides structure for consistency and transparency in reporting. For further information on Nature Research policies, see our Editorial Policies and the Editorial Policy Checklist.

Statistics

For all statistical analyses, confirm that the following items are present in the figure legend, table legend, main text, or Methods section.

n/a	Confirmed	
☐	☒	The exact sample size (*n*) for each experimental group/condition, given as a discrete number and unit of measurement
☐	☒	A statement on whether measurements were taken from distinct samples or whether the same sample was measured repeatedly
☐	☒	The statistical test(s) used AND whether they are one- or two-sided<br>*Only common tests should be described solely by name; describe more complex techniques in the Methods section.*
☐	☒	A description of all covariates tested
☒	☐	A description of any assumptions or corrections, such as tests of normality and adjustment for multiple comparisons
☐	☒	A full description of the statistical parameters including central tendency (e.g. means) or other basic estimates (e.g. regression coefficient) AND variation (e.g. standard deviation) or associated estimates of uncertainty (e.g. confidence intervals)
☐	☒	For null hypothesis testing, the test statistic (e.g. *F*, *t*, *r*) with confidence intervals, effect sizes, degrees of freedom and *P* value noted<br>*Give P values as exact values whenever suitable.*
☒	☐	For Bayesian analysis, information on the choice of priors and Markov chain Monte Carlo settings
☒	☐	For hierarchical and complex designs, identification of the appropriate level for tests and full reporting of outcomes
☒	☐	Estimates of effect sizes (e.g. Cohen's *d*, Pearson's *r*), indicating how they were calculated

Our web collection on statistics for biologists contains articles on many of the points above.

Software and code

Policy information about availability of computer code

Data collection	BD FACSDiva v8.0.2 software
Data analysis	R version 3.4.3 (R foundation, Vienna, Austria); The following R packages: ggalluvial (version 0.12.3) and ggplot2 (version 3.3.3); MEGA7 (https://www.megasoftware.net/); iTOL (version 5); GraphPad Prism (version 8.0.2); FASTQC v0.11.7 (http://www.bioinformatics.babraham.ac.uk/projects/fastqc); bbmap v37.99 (sourceforge.net/projects/bbmap/); MEGAHIT v1.2.9; blastn v2.7.1; FlowJo v10.6.2.; Scripts concerning de novo assembly of HIV-1 genomes are freely available on GitHub: https://github.com/laulambr/virus_assembly. A description of the key operations and instructions on how to install and run the code are provided on the GitHub page. In addition, a test dataset and a description of expected outputs with expected run times are provided.

For manuscripts utilizing custom algorithms or software that are central to the research but not yet described in published literature, software must be made available to editors and reviewers. We strongly encourage code deposition in a community repository (e.g. GitHub). See the Nature Research guidelines for submitting code & software for further information.

Data

Policy information about availability of data

All manuscripts must include a data availability statement. This statement should provide the following information, where applicable:

- Accession codes, unique identifiers, or web links for publicly available datasets
- A list of figures that have associated raw data
- A description of any restrictions on data availability

HIV-1 sequence data that support the findings of this study have been deposited in Genbank with the accession codes MW881651-MW881770 [https://www.ncbi.nlm.nih.gov/nuccore/?term=MW881651%3AMW881770%5Baccn%5D] and MH642355- MH643573 [https://www.ncbi.nlm.nih.gov/nuccore/?term=MH642355%3AMH643573%5Baccn%5D]. External databases used in this study are available online: IMGT® database (IMGT®, the international

ImMunoGeneTics information system®, http://www.imgt.org); McPASTCR database (http://friedmanlab.weizmann.ac.il/McPAS-TCR/); Los Alamos HIV Sequence database (https://www.hiv.lanl.gov); Integration Sites webtool (https://indra.mullins.microbiol.washington.edu/integrationsites); allOnco gene list (http://www.bushmanlab.org/links/genelists). All other data generated or analyzed during this study are included in this paper and its Supplementary Information files. Data underlying main and supplementary figures are provided with this paper as a Source Data file.

Field-specific reporting

Please select the one below that is the best fit for your research. If you are not sure, read the appropriate sections before making your selection.

- [x] Life sciences
- [ ] Behavioural & social sciences
- [ ] Ecological, evolutionary & environmental sciences

For a reference copy of the document with all sections, see nature.com/documents/nr-reporting-summary-flat.pdf

Life sciences study design

All studies must disclose on these points even when the disclosure is negative.

Sample size

This is a descriptive study on 8 participants for which leukapheresis samples were available. The sample size was chosen based on cost and rarity of available samples. Sample size calculations were not performed.

Data exclusions

No data were excluded for the analysis.

Replication

Repetitions of the STIP-Seq protocol were performed for participants P3 (n=2 repetitions), P4 (n=2 repetitions), P5 first (n=5 repetitions), P5 + 3 years (n=2 repetitions), P6 T2 (n=2 repetitions), P7 T2 (n=3 repetitions) and P8 (n=2 repetitions). For all participants, significant overlap in clonal cell populations (HIV proviral sequence, TCR sequence and integration site) was observed between the different repetitions, showing the reproducibility of the approach. Also, clonal p24+ cells (which can be considered as biological replicates) always showed 100% accordance between TCR sequence, near-full length proviral sequence and integration site, validating that these assays produced reproducible results. Overall, all attempts at replication were successful.

Randomization

All participants in the study were part of the same group (HIV+ ART-treated individuals) and allocation into different groups or randomization is not applicable.

Blinding

As this study was not randomized, blinding is not applicable.

Reporting for specific materials, systems and methods

We require information from authors about some types of materials, experimental systems and methods used in many studies. Here, indicate whether each material, system or method listed is relevant to your study. If you are not sure if a list item applies to your research, read the appropriate section before selecting a response.

Materials & experimental systems

n/a	Involved in the study	
[ ]	[x]	Antibodies
[ ]	[x]	Eukaryotic cell lines
[x]	[ ]	Palaeontology and archaeology
[x]	[ ]	Animals and other organisms
[ ]	[x]	Human research participants
[x]	[ ]	Clinical data
[x]	[ ]	Dual use research of concern

Methods

n/a	Involved in the study	
[x]	[ ]	ChIP-seq
[ ]	[x]	Flow cytometry
[x]	[ ]	MRI-based neuroimaging

Antibodies

Antibodies used

p24 KC57-FITC; Dilution 1/500 (Beckman Coulter, Cat. No. 6604665)
p24 28B7-APC; Dilution 1/400 (MediMabs, Cat. No. MM-0289-APC)
CD8-AF700 Clone RPA-T8; Dilution 1/200 (ThermoFisher, Cat. No. 56-0088-41)
CD45RO-BV421 Clone UCHL1; Dilution 1/100 (BD Biosciences, Cat. No. 562649)
CD27-BV605 Clone L128; Dilution 1/100 (BD Biosciences, Cat. No. 562656)
Fixable Viability Stain 510; Dilution 1/1000 (ThermoFisher Scientific, Cat. No. L34957)

Validation

All antibodies are commercially available. Titrations were performed for all antibodies to determine optimal antibody concentrations.
- p24 KC57-FITC (Beckman Coulter): the certificate of analysis can be found on the Beckman Coulter website. It is certified that each batch of p24 KC57 meets the requirements for flow cytometry experiments.
- p24 28B7-APC (MediMabs): validated by Pardons et al. Plos Pathogens 2019
- CD8-AF700 Clone RPA-T8 (ThermoFisher): product data sheet (https://www.thermofisher.com/order/genome-database/generatePdf?productName=CD8a&assayType=PRANT&detailed=true&productId=56-0088-41)

- CD45RO-BV421 Clone UCHL1 (BD Biosciences): reference on BD website (Akbar AN, Terry L, Timms A, Beverley PC, Janossy G. Loss of CD45R and gain of UCHL1 reactivity is a feature of primed T cells. J Immunol. 1988; 140(7):2171-2178)
- CD27-BV605 Clone L128 (BD Biosciences); reference on BD website (Baars PA, Maurice MM, Rep M, Hooibrink B, van Lier RA. Heterogeneity of the circulating human CD4+ T cell population. Further evidence that the CD4+CD45RA-CD27- T cell subset contains specialized primed T cells. J Immunol. 1995; 154(1):17-25)
- Fixable Viability Stain 510 (ThermoFisher Scientific): reference on ThermoFisher Scientific website (Zeng X, Wei YL, Huang J, Newell EW, Yu H, Kidd BA, Kuhns MS, Waters RW, Davis MM, Weaver CT, Chien YH, Immunity (2012))

Eukaryotic cell lines

Policy information about cell lines

Cell line source(s)	ARP-177 Human T-Cell Lymphoma, Jurkat (E6-1) Cells: NIH AIDS Reagent Program<br>ARP-1340 Human Immunodeficiency Virus 1 (HIV-1) Lymphadenopathy-Associated Virus (LAV)-Infected Jurkat E6 Cells (J1.1): NIH AIDS Reagent Program
Authentication	All cell lines were acquired through the NIH AIDS Reagent Program.<br>- ARP-177 Human T-Cell Lymphoma, Jurkat (E6-1) Cells: datasheet can be downloaded here: https://www.hivreagentprogram.org/Catalog/cellBanks/ARP-177.aspx. This cell line was not authenticated at our lab using molecular DNA techniques. However, flow-cytometry analysis confirmed the CD4-positivity of the cell line and the absence of any p24 production.<br>- ARP-1340 Human Immunodeficiency Virus 1 (HIV-1) Lymphadenopathy-Associated Virus (LAV)-Infected Jurkat E6 Cells (J1.1): datasheet can be downloaded here: https://www.hivreagentprogram.org/Catalog/cellBanks/ARP-1340.aspx<br>Cell lines were propagated according to instructions provided by the NIH AIDS Reagent Program. Integration site analysis was performed on the J1.1 cell line, confirming their identity by the recovery of the dominant clonal integration sites.
Mycoplasma contamination	We confirm that all cell lines were negative for mycoplasma.
Commonly misidentified lines<br>(See ICLAC register)	No commonly misidentified cell lines were used in this study.

Human research participants

Policy information about studies involving human research participants

Population characteristics	Eight individuals living with HIV that received successful antiretroviral therapy. Three of the eight participants (P6, P7, P8) underwent an analytical treatment interruption. All participants were males, between the ages of 37 and 66, and infected with a subtype B virus. Participant's characteristics are summarized in Supplementary Table 1.
Recruitment	Participants were not specifically recruited for this study. Cryopreserved PBMCs, collected by leukapheresis, were used in this study. Participants were previously recruited at two clinical centres: McGill University Health Centre, Montréal QC, Canada; Universitair Ziekenhuis Gent, Gent, Belgium. Participants were recruited on an opt-in basis. As all participants were deemed representative for the population of HIV-1+ individuals in the respective hospitals, self-selection bias, or other biases should not have impacted the results of this study.
Ethics oversight	All participants were adults and signed informed consent forms approved by the Ethics Committee of the Ghent University Hospital (Belgium), McGill University Health Centre and Centre Hospitalier de l'Université de Montréal (Canada).

Note that full information on the approval of the study protocol must also be provided in the manuscript.

Flow Cytometry

Plots

Confirm that:

- [x] The axis labels state the marker and fluorochrome used (e.g. CD4-FITC).
- [x] The axis scales are clearly visible. Include numbers along axes only for bottom left plot of group (a 'group' is an analysis of identical markers).
- [x] All plots are contour plots with outliers or pseudocolor plots.
- [x] A numerical value for number of cells or percentage (with statistics) is provided.

Methodology

Sample preparation	CD4 T cells were isolated from PBMC by negative magnetic selection using the EasySep Human CD4 T Cell Enrichment Kit (StemCell Technology, 19052). Purity was typically >98%. 5-10x106 CD4 T cells were resuspended at 2x106 cells/mL in RPMI + 10% Fetal Bovine Serum (FBS, HyClone RB35947) and antiretroviral drugs were added to the culture (200nM raltegravir, 200nM lamivudine) to avoid new cycles of replication. Cells were stimulated with 1μg/mL ionomycin (Sigma, I9657) and 162nM PMA (Sigma, P8139). After a 24h-stimulation, a maximum of 10x106 cells per condition were resuspended in PBS and stained with fixable viability stain 510 for 20 min at RT. Cells were then stained with antibodies against cell surface molecules (CD8, CD45RO, CD27) in PBS + 2% FBS for 20min at 4°C. After a 5 min-centrifugation step at 4°C to pre-chill the cells, CD4 cells were vortexed to avoid clumping and 1mL of ice-cold methanol (-20°C) was gently added. Cells were fixed/permeabilized

in methanol for 15 min on ice. Intracellular p24 staining was performed in PBS + 2% FBS using a combination of 2 antibodies (p24 KC57-FITC, p24 28B7-APC) (45min, RT). Cells were then washed and resuspended in PBS for subsequent sorting. In all experiments, CD4 T cells from an HIV-negative control were included to set the threshold of positivity. The detailed protocol of the methanol-based HIV-Flow procedure can be found here: https://protocols.io/view/methanol-based-hiv-flow-bpedmja6.

Instrument	BD FACSAria Fusion
Software	Data collection: BD FACSDiva v8.0.2 software<br>Data analysis: FlowJo v10.6.2
Cell population abundance	The frequency of p24 double-positive cells (KC57-FITC+ ,28B7-APC+) ranged from 0.8 to 9.5 cells / million CD4 T cells.
Gating strategy	All the relevant gating strategies are described in Supplementary Fig. 9. For each experiment, CD4 T cells from an HIV-negative control sample were used to set the threshold of positivity for p24+ cells (KC57-FITC+ ,28B7-APC+).

☒ Tick this box to confirm that a figure exemplifying the gating strategy is provided in the Supplementary Information.
